# Proposed Mechanism of Long-Term Intraocular Pressure Lowering With the Bimatoprost Implant

**DOI:** 10.1167/iovs.64.3.15

**Published:** 2023-03-06

**Authors:** W. Daniel Stamer, Kristin M. Perkumas, Min H. Kang, Mohammed Dibas, Michael R. Robinson, Douglas J. Rhee

**Affiliations:** 1 Department of Ophthalmology, Duke University, Durham, North Carolina, United States; 2 Department of Ophthalmology & Visual Sciences, University Hospitals, Case Western Reserve University, Cleveland, Ohio, United States; 3 Allergan, an AbbVie company, Irvine, California, United States

**Keywords:** biodegradable implant, eye bank, extracellular matrix metabolism (ECM), prostaglandin analog, mechanism of action

## Abstract

**Purpose:**

The purpose of this study was to evaluate the effects of pharmacologically relevant bimatoprost and bimatoprost free acid (BFA) concentrations on matrix metalloproteinase (MMP) gene expression in cells from human aqueous outflow tissues.

**Methods:**

MMP gene expression by human trabecular meshwork (TM), scleral fibroblast (SF), and ciliary muscle (CM) cells exposed to 10 to 1000 µM bimatoprost or 0.1 to 10 µM BFA (intraocular concentrations after intracameral bimatoprost implant and topical bimatoprost dosing, respectively) was measured by polymerase chain reaction array.

**Results:**

Bimatoprost dose-dependently upregulated MMP1 and MMP14 mRNA in all cell types and MMP10 and MMP11 mRNA in TM and CM cells; in TM cells from normal eyes, mean MMP1 mRNA levels were 62.9-fold control levels at 1000 µM bimatoprost. BFA upregulated MMP1 mRNA only in TM and SF cells, to two- to three-fold control levels. The largest changes in extracellular matrix (ECM)-related gene expression by TM cells derived from normal (*n* = 6) or primary open-angle glaucoma (*n* = 3) eyes occurred with 1000 µM bimatoprost (statistically significant, ≥50% change for 9–11 of 84 genes on the array, versus 1 gene with 10 µM BFA).

**Conclusions:**

Bimatoprost and BFA had differential effects on MMP/ECM gene expression. Dramatic upregulation in MMP1 and downregulation of fibronectin, which occurred only with bimatoprost at high concentrations observed in bimatoprost implant–treated eyes, may promote sustained outflow tissue remodeling and long-term intraocular pressure reduction beyond the duration of intraocular drug bioavailability. Variability in bimatoprost-stimulated MMP upregulation among cell strains from different donors may help explain differential long-term responses of patients to bimatoprost implant.

##  

Primary open-angle glaucoma (POAG) is associated with changes in extracellular matrix (ECM) components in the trabecular meshwork (TM) and other structural changes in the TM that cause increased resistance to aqueous humor outflow and result in increased intraocular pressure (IOP).^1^^,^^2^ First-line treatment of POAG is usually a topical ophthalmic medication to lower IOP, and the prostaglandin analog/prostamide (PGA) class of topical IOP-lowering medications (e.g., bimatoprost, latanoprost, tafluprost, and travoprost) is commonly used because these medications are well tolerated, systemically safe, and the most efficacious in lowering IOP.^3^^,^^4^ However, adherence of patients to topical IOP-lowering therapy is poor. In a study using pharmacy claims data for 3310 patients with glaucoma, on average, patients who filled prescriptions for a topical PGA had medication available for dosing only 37% of the days in the year.^5^ Additionally, in a retrospective, longitudinal study involving 1234 newly diagnosed and treated patients with POAG, only an estimated 15% of patients had persistently good adherence through 4 years of treatment.^6^

Bimatoprost implant (Durysta; Allergan, an AbbVie company, North Chicago, IL, USA) is a biodegradable, intracameral, sustained-release implant that was developed to improve adherence to treatment in glaucoma and reduce IOP without the need for daily eye drops.^7^ The implant contains 10 µg bimatoprost in a drug delivery system consisting of biodegradable polymers (similar to those in biodegradable sutures).^8^ After intracameral administration, the implant slowly releases bimatoprost to lower IOP as the polymers are hydrolyzed and metabolized to carbon dioxide and water.^8^ The implant was designed to release bimatoprost at a steady rate for 3 to 4 months.^8^

Pharmacokinetic studies using normotensive beagle dogs have shown that administration of a 15-µg bimatoprost implant achieves drug concentrations in the iris-ciliary body (a target tissue for IOP lowering) 4400-fold higher than those obtained with once-daily topical bimatoprost 0.03% eye drops.^9^ After topical bimatoprost 0.03% dosing, the major drug moiety detected in the iris-ciliary body was bimatoprost acid, at a low nanomolar concentration, whereas after bimatoprost implant administration, the major drug moiety detected in the iris-ciliary body was the intact bimatoprost molecule, at a concentration in the mid micromolar range.^9^ At 4.2 months after bimatoprost implant administration, intraocular drug levels in the dog model were beneath the limit of detection.^8^ Consistent with these results, assays in vitro showed complete drug release within 90 days, and drug levels were below the limit of detection (<0.05 ng/mL) in aqueous humor samples taken from 2 patients at 3 to 4 months after their last implant administration.^8^

Interestingly, the effects of the bimatoprost implant on IOP typically last beyond the period of drug release and intraocular drug bioavailability. In a phase I/II clinical study, 28% (21/75) of patients with open-angle glaucoma required no additional IOP-lowering treatment for up to 2 years after a single administration of 6-, 10-, 15-, or 20-µg bimatoprost implant.^10^ Further, in the phase III ARTEMIS 1 and ARTEMIS 2 studies, patients with open-angle glaucoma or ocular hypertension received 3 administrations of 10- or 15-µg bimatoprost implant at intervals of 16 weeks, and IOP was controlled in the majority of patients without additional treatment at 1 year after the third implant administration.^8^^,^^11^

The mechanism for sustained IOP lowering after bimatoprost implant administration has not been determined. However, topical PGAs reduce IOP in humans by increasing aqueous outflow primarily through the unconventional (uveoscleral) and secondarily through the conventional (TM) pathways, and there is abundant evidence that the molecular mechanism for the increase in aqueous outflow involves PGA-induced upregulation of matrix metalloproteinases (MMPs) and an increase in MMP-mediated turnover of the ECM in the uveoscleral and TM outflow pathways.^8^^,^^12^ The MMPs are a large family of zinc-dependent proteolytic enzymes that degrade various components of the ECM, including collagens, elastin, fibronectin, and other matrix glycoproteins and proteoglycans, and have an essential role in tissue remodeling.^13^ In studies using primates, topical PGA treatment produced changes in the extracellular material within the ciliary muscle^14^ and upregulated MMPs in association with the reduction in IOP.^15^ Further, long-term topical PGA treatment of primates was shown to lead to tissue remodeling in the ciliary muscle and TM (increased spaces between muscle bundles in the anterior ciliary muscle, expansion of the juxtacanalicular region, and loss of ECM in the TM) that could potentially reduce aqueous outflow resistance.^16^ Other studies using cell cultures derived from ocular tissues have shown that PGA-induced upregulation of MMPs is dependent on both the identity and concentration of the PGA.^17^^–^^21^

Due to substantial effects on ECM turnover and its importance in outflow regulation, we hypothesized that long-term IOP lowering after bimatoprost implant administration is due to prolonged and amplified upregulation of MMPs induced by the continuous and higher drug levels in target tissues, leading to more durable outflow tissue remodeling and sustained effects on IOP.^22^ In this study, to test our hypothesis and help explain the long-term IOP lowering with bimatoprost implant, we evaluated the effects of pharmacologically relevant bimatoprost and bimatoprost free acid (BFA) concentrations on MMP and ECM gene and protein expression in cells from human outflow tissues.

## Methods

This experimental study evaluated changes in MMP gene and protein expression stimulated by bimatoprost and BFA in TM, ciliary muscle (CM), and scleral fibroblast (SF) cells derived from human cadaver eyes. The study was approved by the Institutional Review Board of the Duke University Medical Center and was conducted in accordance with the Helsinki Declaration of 1964 and its later amendments.

### Cell Culture

Cadaver eyes (17 normal, 3 with POAG) from 20 separate individuals, ages 3 months to 91 years, were obtained from Advancing Sight (Alabama Eye Bank) and Miracles in Sight (North Carolina Eye Bank) within 6 hours postmortem, and TM, CM, or SF cells were isolated from each eye. Characteristics of the donor eyes and cell strains are listed in Supplementary Table S1.

TM tissue was dissected and TM cells were isolated^23^ and characterized,^24^ as described previously. TM cells were grown on gelatin-coated plates at 37°C under 5% CO_2_ in low glucose Dulbecco's modified Eagle medium (DMEM) with L-glutamine, 110 mg/mL sodium pyruvate, 10% fetal bovine serum (FBS; Bio-Techne, Minneapolis, MN, USA), 100 U/mL penicillin, and 100 µg/mL streptomycin (pen/strep).

Ciliary body tissue was dissected, and CM cells were cultured from explants and characterized as described previously.^25^^,^^26^ The cultures were grown on uncoated plates at 37°C under 5% CO_2_ in DMEM with L-glutamine, 20% FBS, and 100 µg/mL gentamicin.

SF cells were derived from scleral limbal tissue away from potential TM or CM contaminants. The scleral tissue was dissected, and explants were allowed to grow undisturbed on uncoated plates at 37°C under 5% CO_2_ in high glucose DMEM with L-glutamine, 110 mg/mL sodium pyruvate, 10% FBS, and pen/strep until confluence was reached. Cells were subcultured into larger plates and grown in the same medium.

### Gene Expression Assays and Analysis

For all cell types, cells from passages 4 to 7 were used for assays. Cells were cultured in uncoated 6-well plates until confluent. The concentration of FBS in the medium of the TM cells was reduced to 1% for 1 week before the drug treatment. The cells were treated for 24 hours with bimatoprost (10 µM, 100 µM, or 1000 µM), BFA (0.1 µM, 1 µM, or 10 µM), or vehicle control (1% ethanol) in DMEM containing 1% FBS and pen/strep. One well of cells was used for each cell strain and drug concentration. To mimic glaucomatous conditions in some experiments, cells were treated with 2.5 ng/mL human recombinant transforming growth factor-beta 2 (TGF-β2; R & D Systems, Minneapolis, MN, USA) or vehicle control for 72 hours, with bimatoprost, BFA, or vehicle control added for the last 24 hours.

Following the 24-hour treatment period with PGA or vehicle, expression of 84 genes related to the ECM was measured on real-time polymerase chain reaction (PCR) array with the Human Extracellular Matrix and Adhesion Molecules RT^2^ Profiler PCR array (Qiagen, Germantown, MD, USA). The genes on this array (Supplementary Table S2) include collagen and other ECM constituents, MMPs and their inhibitors, integrins and other cell adhesion molecules, and other proteins important for cell-ECM interactions. The medium was removed from the cells, RNA was isolated from cell lysates using the RNeasy Kit (Qiagen, Hilden, Germany), and cDNA was prepared with the RT^2^ First Strand Kit (Qiagen), mixed with RT^2^ SYBR Green qPCR Mastermix (Qiagen), and aliquoted into the wells of the array. A CFX96 Touch Real-Time PCR machine (Bio-Rad, Hercules, CA, USA) was used for the PCR amplification and fluorescence signal detection. The threshold cycle (CT) for each well was determined by the cycler software, and relative gene expression levels were determined using web-based tools of the Qiagen GeneGlobe Data Analysis Center (https://geneglobe.qiagen.com/us/analyze). Housekeeping genes on the PCR array (beta actin, beta-2-microglobulin, glyceraldehyde-3-phosphate dehydrogenase, hypoxanthine phosphoribosyltransferase 1, and 60S ribosomal protein P0) were used for normalization of the real-time PCR data. Fold gene expression levels in PGA-treated cells relative to those in vehicle-treated control cells were determined using the 2^−∆∆CT^ method, and statistically significant differences in normalized gene expression levels (mean ∆CT values) between PGA-treated and vehicle-treated control cells were analyzed with t-tests and an alpha level of 0.05.

### MMP1 Protein Secretion Assays and Analysis

TM and CM cells were treated for 24 hours with bimatoprost (10 µM, 100 µM, and 1000 µM), BFA (0.1 µM, 1 µM, and 10 µM), or vehicle control as described for the gene expression assays. The medium was then removed from the cells, and levels of MMP1 protein in the medium were measured with an enzyme-linked immunosorbent assay (ThermoFisher Scientific, Waltham, MA, USA).

### Immunocytochemistry

TM cells were plated on glass coverslips in a 6-well plate and when they reached 100% confluence the medium was switched to DMEM with 1% FBS for 4 days. Cells were then treated with BIM (1000 µM) or vehicle for 24 hours prior to fixing with 4% paraformaldehyde for 10 minutes. Following 3 washes with phosphate-buffered saline (PBS), cells on coverslips were blocked in Cell Signaling Blocking Buffer (PBS with 5% goat serum and 0.3% Triton X-100) for 1 hour. Cells on coverslips were then incubated overnight at 4°C with anti-fibronectin IgGs conjugated to Alexa Fluor 488 (Cell Signaling) in Cell Signaling Antibody dilution buffer (PBS with 1% BSA and 0.3% Triton X-100). Following three washes with PBS, cells on coverslips were DAPI stained then mounted onto slides with Immu-Mount (Epredia, Portsmouth, NH, USA) and sealed with nail polish. Treated and control cells were imaged using confocal microscopy during the same session, at identical settings.

### Fibronectin ELISA Assay

Conditioned media was collected from TM cells treated with vehicle or BIM (1000 µM) for 24 hours and immediately frozen at −80°C. Frozen media samples were thawed on ice and brought to room temperature. Media samples were diluted 1:500 in assay buffer following the protocol for the Fibronectin Elisa Assay (ThermoFisher Scientific) to fall within the standard curve. Briefly, diluted media samples (50 µL) were added to wells of a 96-well plate precoated with biotin-conjugated anti human fibronectin antibody, in duplicate. Samples were incubated on the plate for 2 hours, followed by washing. Streptavidin-horseradish peroxidase was added to each well, followed again by washing. Substrate (tetramethyl-benzidine) was added for color development. The reaction was stopped with 1 M phosphoric acid, then was read at 450 nm on a plate reader. The standards were plotted using a five-parameter curve fit, and the concentration of fibronectin in the samples was calculated.

### Cell Viability Assay

Cells were cultured on uncoated 6-well plates until confluent in DMEM containing 10% FBS, then were switched to DMEM containing 1% FBS. After 1 week, cells were treated with 2 mL DMEM containing implant levels of bimatoprost (10–1000 µM) or vehicle for 24 hours. As a positive control, one well from 3 different TM cell strains was treated with 1% Triton X-100 to lyse all of the cells 30 minutes prior to media collection. Media was collected from all wells and frozen at −80°C. ToxiLight bioassay kit (Lonza) was used to measure the release of adenylate kinase (AK) from damaged cells. Frozen conditioned media, AK detection agent, and AK buffer were brought to room temperature. Detection agent was added to buffer (10 mL), mixed, and incubated at room temperature. After 15 minutes, 100 µL of mixture was added to 20 µL of conditioned media from samples (in triplicate) in a 96-well white/opaque bottom plate while maintained in dark conditions. After 5 minutes, plate luminescence was measured.

## Results

The ranges of bimatoprost and BFA concentrations used for the treatment of the TM, CM, and SF cells were based on results from a pharmacokinetics study of drug distribution after bimatoprost implant and bimatoprost eye drop administration in beagle dogs.^9^ After intracameral administration of a 15-µg bimatoprost implant in beagle dogs, the mean maximal drug concentration (*C*_max_) measured in the iris–ciliary body target tissue was 44 µM for the intact bimatoprost molecule and 494 nM for BFA (Supplementary Fig. S3). In humans, mean *C*_max_ values after bimatoprost implant administration are predicted to be 2 to 3 times higher (approximately 100 µM for bimatoprost and 1 µM for BFA) because the anterior chamber volume in human eyes is approximately 20% that in dog eyes, and the resulting greater drug concentration is only partially offset by a lower flow rate in human eyes^27^ and the lower clinical dose. To take into account the variability in measured *C*_max_ values among dogs, which is also likely to exist among humans, we bracketed these concentrations and tested a 3-log unit range of drug concentrations when evaluating the effects of bimatoprost and BFA on gene expression by human TM, CM, and SF cells, using concentrations of 10 µM, 100 µM, and 1000 µM bimatoprost and 0.1 µM, 1 µM, and 10 µM BFA. These drug concentrations tested in the gene expression assays reflect bimatoprost concentrations that could be expected in human eyes after bimatoprost implant administration and BFA concentrations that could be expected in human eyes after either bimatoprost implant or topical bimatoprost administration.

**Figure 1.**
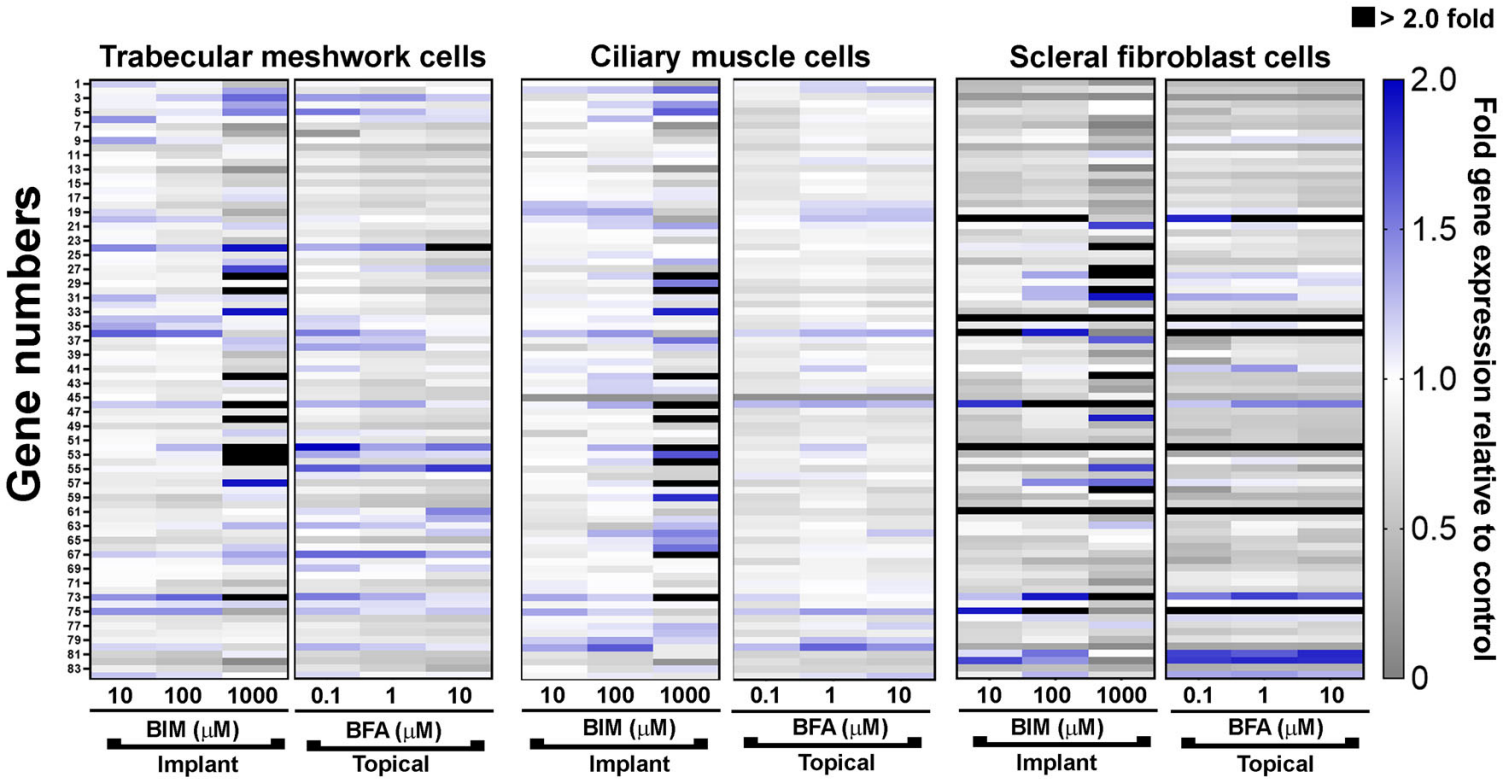
Heat map of the mean expression levels of 84 extracellular matrix–related genes in response to treatment for 24 hours with bimatoprost implant– or topical bimatoprost–relevant concentrations of bimatoprost (BIM; 10 µM, 100 µM, or 1000 µM) or bimatoprost free acid (BFA; 0.1 µM, 1 µM, or 10 µM), respectively, relative to control (vehicle) treatment. Responses in individual trabecular meshwork (*n* = 6), ciliary muscle (*n* = 6), and scleral fibroblast (*n* = 3–4) cell strains were independently tested, then averaged. Expression levels are indicated by colors, where *grays* indicate a decrease, *white* indicates no change, and *blue* and *black* indicate an increase in gene expression relative to control.

We observed that the effects of bimatoprost and BFA treatment for 24 hours on the expression of ECM-related genes in TM, CM, and SF cells derived from normal human eyes were dependent on the PGA form (bimatoprost versus BFA), its concentration, and the cell type (Fig. 1). For all three cell types, the number of genes with significantly changed expression versus control was highest with the highest concentration of bimatoprost tested (1000 µM). At this concentration, we observed no cytotoxicity (Supplementary Fig. S4). Nine genes in TM cells treated with 1000 µM bimatoprost (Table 1) and 7 genes in CM cells treated with 1000 µM bimatoprost (Table 2) on the array of 84 genes showed a statistically significant change in expression of at least 50% from the control, compared with one gene in TM cells and no genes in CM cells treated with 10 µM BFA. Because of less variability in drug effects among different SF cell strains, a larger number of significant changes in gene expression versus control was observed in SF cells treated with bimatoprost and BFA (Table 3). The majority of these changes in SF cells were decreases in gene expression.

**Table 1.** Statistically Significant (*P* < 0.05) Changes in Gene Expression of at Least 50% in TM Cell Strains Exposed to Bimatoprost and BFA (*n* = 6)

	Mean Fold Gene Expression Relative to Control	
	Bimatoprost	BFA
Gene	1000 µM	1 µM
CNTN1	0.19	
COL1A1	0.15	
ICAM1	4.79	
ITGA2	2.65	
ITGA5	1.93	
ITGB3	2.43	
LAMA3	3.82	
MMP11	2.78	
MMP14	1.92	
NCAM1		0.50
Total^*^	9	1

* Number of genes with a statistically significant, ≥50% increase or decrease in expression relative to control.

BFA, bimatoprost free acid; TM, trabecular meshwork.

**Table 2.** Statistically Significant (*P* < 0.05) Changes in Gene Expression of at Least 50% in CM Cell Strains Exposed to Bimatoprost and BFA (*n* = 6)

	Mean Fold Gene Expression Relative to Control
	Bimatoprost
Gene	1000 µM
ADAMTS1	0.44
ITGA2	2.90
ITGAL	1.56
LAMA3	2.92
MMP10	1.67
MMP14	2.64
PECAM1	1.56
Total^*^	7

* Number of genes with a statistically significant, ≥50% increase or decrease in expression relative to control.

BFA, bimatoprost free acid; CM, ciliary muscle.

**Table 3.** Statistically Significant (*P* < 0.05) Changes in Gene Expression of at Least 50% in SF Cell Strains Exposed to Bimatoprost and BFA (*n* = 3–4)

	Mean Fold Gene Expression Relative to Control					
	Bimatoprost			BFA		
Gene	10 µM	100 µM	1000 µM	0.1 µM	1 µM	10 µM
ADAMTS13	0.49					0.47
ADAMTS8	0.20	0.17	0.09		0.19	0.16
CNTN1			0.02			
COL14A1	0.39	0.43	0.24	0.44	0.43	0.35
COL1A1			0.03			
COL5A1			0.20			
COL6A1			0.41			
COL7A1			0.28			0.49
CTGF		2.35			2.22	
CTNNA1			1.74			
FN1	0.46					
ICAM1			4.43			
ITGA4			0.32			
ITGB3			3.10			
ITGB4	0.49		0.41			
ITGB5			0.27			
LAMA1			6.91			
LAMB1			0.41			
LAMC1			0.50			
MMP11			0.43			
MMP14			1.57			
MMP16	0.42	0.48		0.41	0.41	0.42
SELE				0.47		
SELL		0.41	0.38	0.31		0.41
SPARC			0.19			
THBS1			0.14			
TIMP2			0.45			
TIMP3	0.34	0.31	0.09	0.31	0.32	0.30
VCAN	0.47		0.37	0.36	0.42	0.40
Total^*^	8	6	24	6	6	8

* Number of genes with a statistically significant, ≥50% increase or decrease in expression relative to control.

BFA, bimatoprost free acid; SF, scleral fibroblast.

Across all cell types, the most substantial changes in ECM-related and MMP gene expression occurred with 1000 µM bimatoprost. Bimatoprost dose-dependently upregulated MMP1 (collagenase) mRNA in all cell types. Among cell strains isolated from normal eyes, the greatest fold increase in MMP1 gene expression (to 62.9-fold the levels in vehicle-treated control cells) occurred in TM cells treated with 1000 µM bimatoprost. In contrast, BFA upregulated MMP1 mRNA only in TM and SF cells, and only to levels two- to three-fold those in control cells, with no dose response. Bimatoprost, but not BFA, also dose-dependently upregulated MMP10, MMP11, and MMP14 gene expression in TM and CM cells (see Fig. 1). MMP14 gene expression by SF cells was also significantly increased by treatment with 1000 µM bimatoprost. Gene expression of collagen type 1 alpha 1 chain (COL1A1) and vascular cell adhesion molecule 1 (VCAM1) were dose dependently reduced by bimatoprost in TM, CM, and SF cells (see Fig. 1).

**Figure 2.**
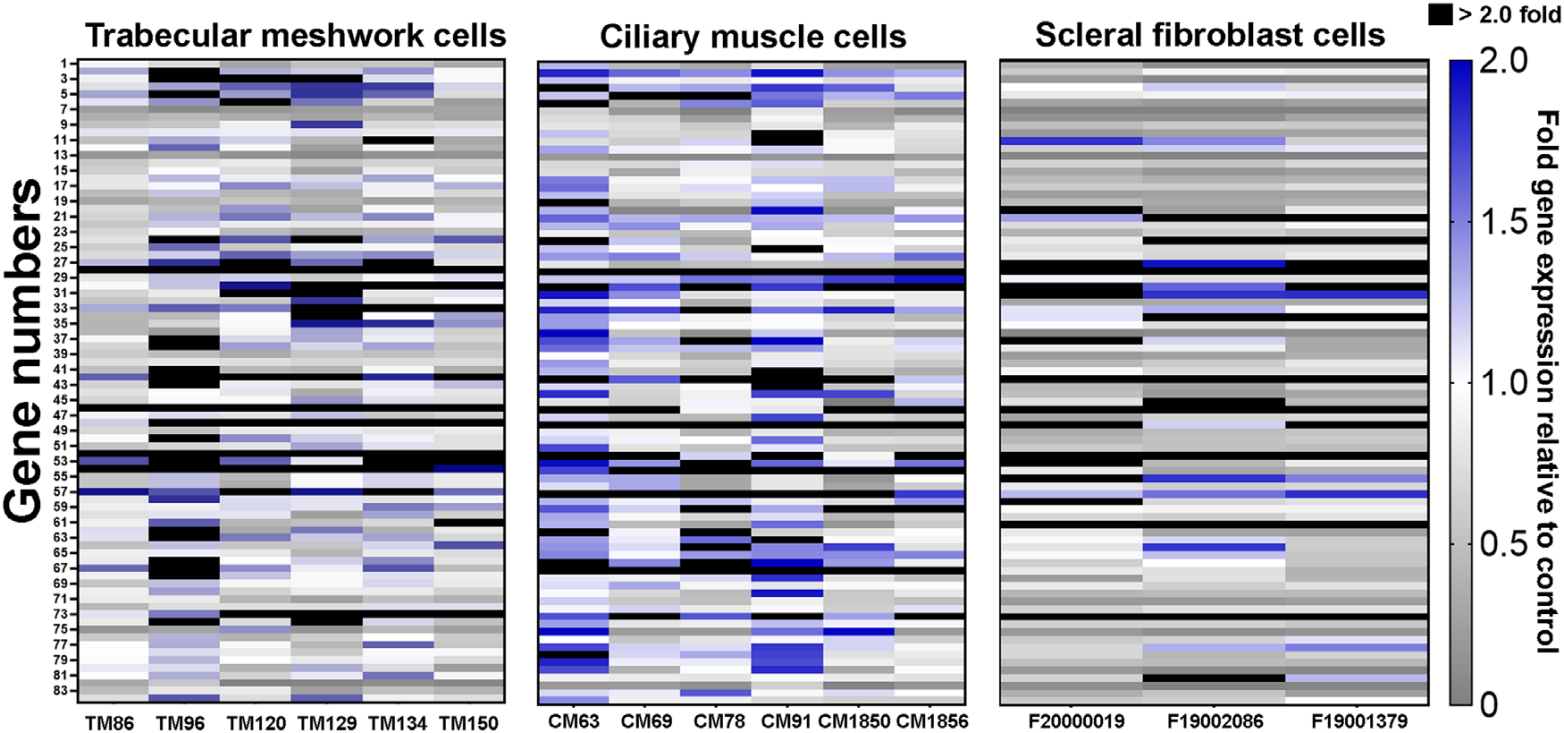
Heat map of the expression levels of 84 extracellular matrix–related genes in individual trabecular meshwork (*n* = 6), ciliary muscle (*n* = 6), and scleral fibroblast (*n* = 3) cell strains in response to treatment with 1000 µM bimatoprost for 24 hours. Expression levels are indicated by colors, where *grays* indicate a decrease, *white* indicates no change, and *blue* and *black* indicate an increase in gene expression relative to control.

Variability in the bimatoprost- and BFA-stimulated changes in the expression of ECM-related genes was evaluated among cell strains from different donors. For each cell type, large variability in the response to 1000 µM bimatoprost was observed among cell strains (Fig. 2). Treatment with 1000 µM bimatoprost led to increased expression of integrin-beta 3 (ITGB3), laminin-alpha 3 (LAMA3), and MMP14 mRNA in all TM, CM, and SF cell strains and increased expression of intercellular adhesion molecule 1 (ICAM1), integrin-alpha 5 (ITGA5), and MMP11 mRNA in all TM and CM cell strains (see Fig. 2). In addition, at least 5 of the 6 TM and CM cell strains demonstrated increased expression of ADAM metallopeptidase with thrombospondin type 1 motif 13 (ADAMTS13), CD44 molecule Indian blood group (CD44), integrin-alpha 2 (ITGA2), MMP1, and MMP10 mRNA when treated with 1000 µM bimatoprost (see Fig. 2). Expression of the laminin-alpha 1 (LAMA1) and MMP1 genes was consistently increased to levels greater than 2-fold those in control cells in all TM cell strains. Comparisons of the dose-response to bimatoprost and BFA among cell strains confirmed large variability in the responses to both bimatoprost and BFA among the TM cell strains (Supplementary Fig. S5), CM cell strains (Supplementary Fig. S6), and SF cell strains (Supplementary Fig. S7). In all cell types, the highest concentration of bimatoprost used had the most significant effects on gene expression.

**Figure 3.**
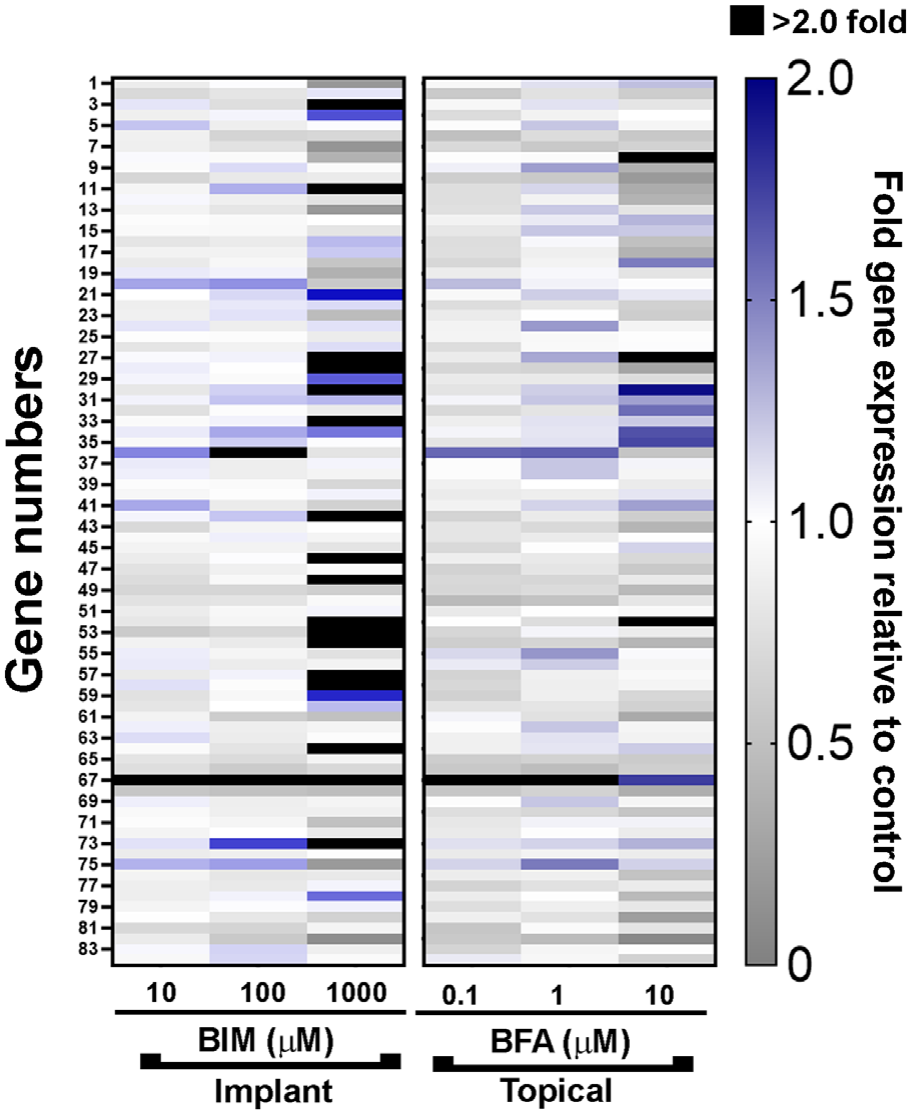
Gene expression in trabecular meshwork cells derived from donor eyes with primary open-angle glaucoma. Heat map of the mean expression levels of 84 extracellular matrix–related genes in response to treatment for 24 hours with bimatoprost implant– or topical bimatoprost–relevant concentrations of bimatoprost (BIM; 10 µM, 100 µM, or 1000 µM) or bimatoprost free acid (BFA; 0.1 µM, 1 µM, or 10 µM), respectively, relative to control (vehicle) treatment. Responses in three individual trabecular meshwork cell strains were independently tested, then averaged. Expression levels are indicated by colors, where *grays* indicate a decrease, *white* indicates no change, and *blue* and *black* indicate an increase in gene expression relative to control.

**Figure 4.**
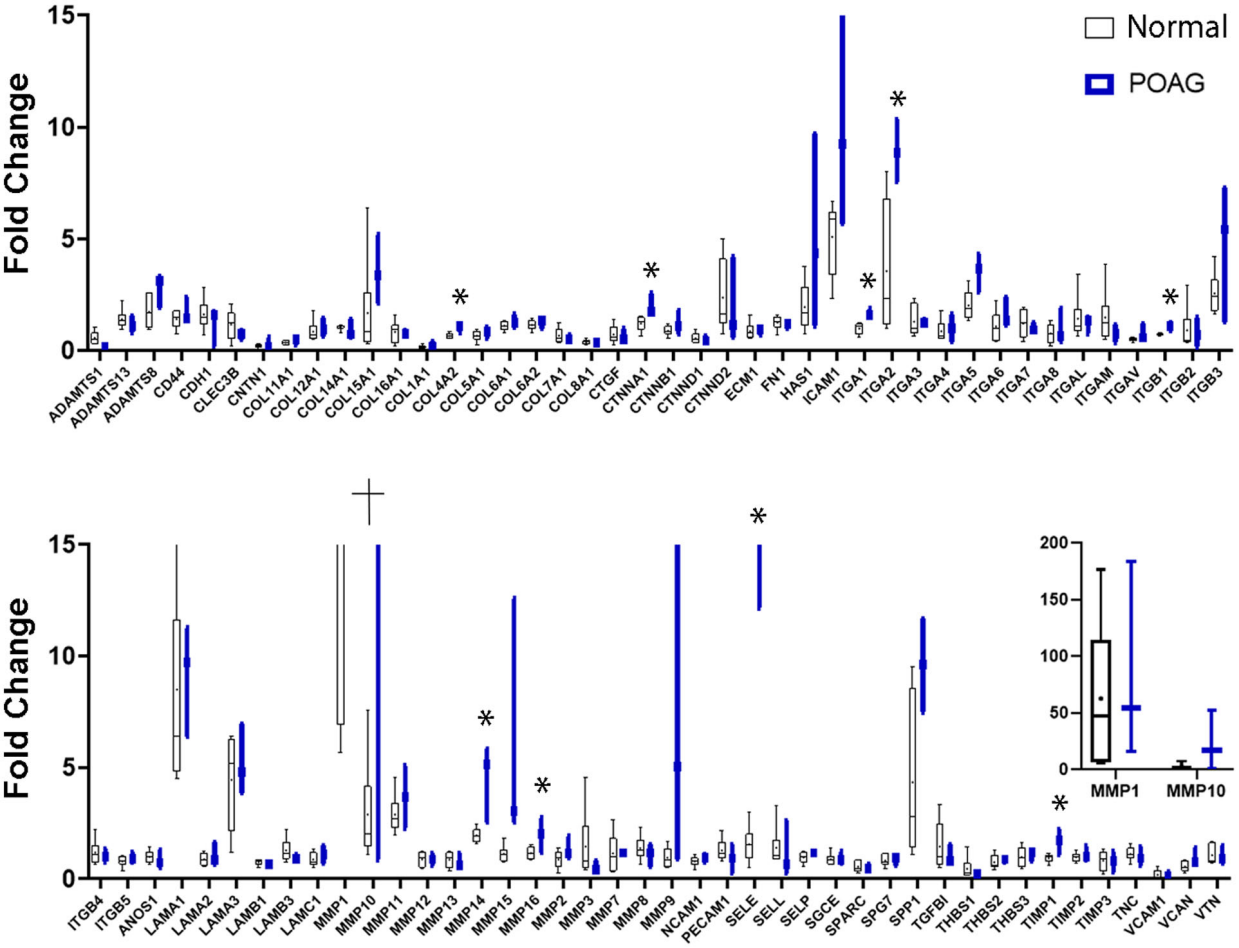
Gene expression changes in TM cell strains derived from normal donor eyes and donor eyes with POAG in response to treatment with a bimatoprost implant–relevant concentration of bimatoprost (1000 µM) for 24 hours. Gene expression is expressed as fold level relative to vehicle-treated control cells. Box and whisker plots in *black* show the response by normal TM cell strains (*n* = 6) with the *line* and *dot* in the box indicating the median and mean, respectively, the *top* and *bottom* edges of the box indicating the 25th and 75th percentiles, and the whiskers extending to the most extreme data points. Data shown in *blue* are the median and range of gene expression in the TM cell strains (*n* = 3) derived from glaucomatous eyes. The inset shows the data with expanded *y*-axis for 2 genes, MMP1 and MMP10 (dagger on bottom panel), which showed a mean fold change of >15 fold. *P < 0.05 versus normal TM cell strains. MMP, matrix metalloproteinase; POAG, primary open-angle glaucoma; TM, trabecular meshwork.

The cell strains used in the assays above, and those used in the literature, were derived from normal donor eyes. To evaluate a potential relationship between glaucoma and the effects of PGAs on MMP gene expression, we established cultures of three additional TM cell strains from donor eyes with POAG. Similar to the findings in the TM cell strains from normal donors, the most substantial changes in ECM-related gene expression were in response to treatment with the 1000 µM concentration of bimatoprost (see Fig. 3). The pattern of changes in gene expression was generally similar in the POAG and normal TM cell strains, with the 1000 µM concentration of bimatoprost producing significant increases in MMP14, ICAM1, ITGA2, ITGA5, and LAMA3 mRNA levels, as well as significant decreases in ADAMTS1, contactin-1 (CNTN1), and catenin-delta 1 (CTNND1) mRNA levels, in TM cells derived from POAG eyes (Table 4) as well TM cells derived from normal eyes (see Table 1). However, there were differences between the POAG and normal TM cell strains with respect to the statistical significance of some changes in gene expression. For example, the dose-dependent mean increase in MMP11 gene expression did not reach statistical significance in the POAG TM cell strains, and the mean increase in SSP1, ADAMTS8, and catenin-alpha 1 (CTNNA1) gene expression after exposure to 1000 µM bimatoprost was significant in the POAG TM cell strains, but not the normal TM cell strains. As in the normal TM cell strains, the largest change in gene expression in response to 1000 µM bimatoprost in the POAG cell strains was for MMP1 (see Fig. 4). MMP1 mRNA levels were upregulated 84.8-fold in the POAG cell strains by 1000 µM bimatoprost. Analysis of the pooled normal and POAG TM cell strains showed a statistically significant, 73.85-fold upregulation of MMP1 gene expression by the 1000 µM concentration of bimatoprost (*P* = 0.018).

**Table 4.** Statistically Significant (*P* < 0.05) Changes in Gene Expression in TM Cell Strains Derived From Eyes With POAG Exposed to Bimatoprost and BFA (*n* = 3)

	Mean Fold Gene Expression Relative to Control		
	Bimatoprost	BFA	BFA
Gene	1000 µM	1 µM	10 µM
ITGB1		0.86	
HAS1			2.83
ADAMTS1	0.18		
ADAMTS8	2.03		
CNTN1	0.16		
CTNNA1	1.94		
CTNND1	0.47		
ICAM1	12.44		
ITGA1	1.64		
ITGA5	3.46		
LAMA3	5.03		
MMP14	4.21		
SPP1	9.43		
Total^*^	11	1	1

* Number of genes with a statistically significant increase or decrease in expression relative to control.

BFA, bimatoprost free acid; POAG, primary open-angle glaucoma; TM, trabecular meshwork.

**Figure 5.**
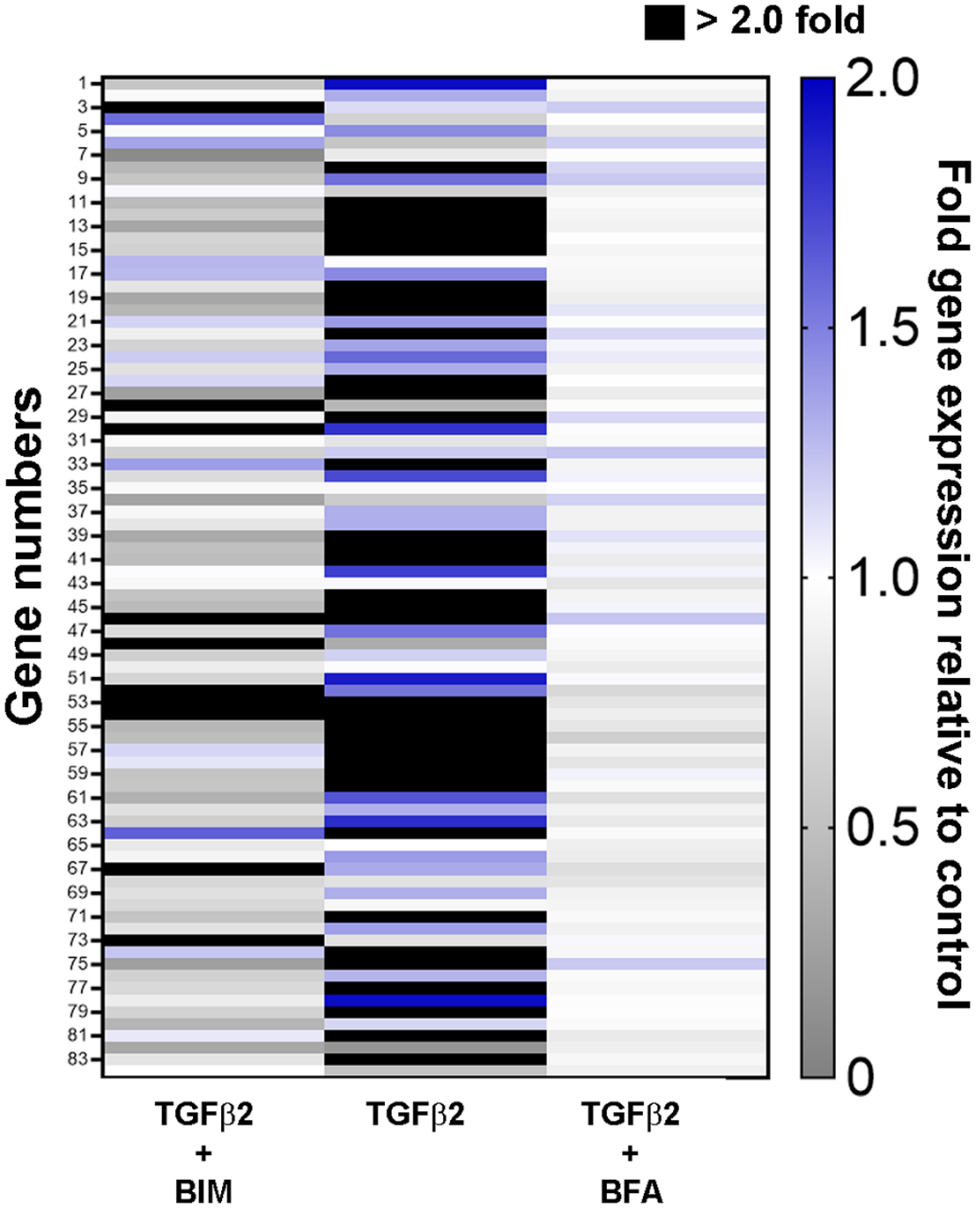
Effects of bimatoprost (BIM) and bimatoprost free acid (BFA) on gene expression in trabecular meshwork (TM) cells treated with transforming growth factor-beta 2 (TGF-β2). Heat map of the mean expression levels of 84 extracellular matrix–related genes in response to treatment for 24 hours with 2.5 ng/mL TGF-β2 in the presence or absence of 1000 µM BIM and 10 µM BFA. Expression levels for TGF-β2 are expressed relative to no TGF-β2 (vehicle control); expression levels for TGF-β2 + BIM and TGF-β2 + BFA are expressed relative to TGF-β2 alone (with vehicle control for BIM and BFA). Responses in individual trabecular meshwork (*n* = 6) cell strains were independently tested, then averaged. Expression levels are indicated by colors, where *grays* indicate a decrease, *white* indicates no change, and *blue* and *black* indicate an increase in relative gene expression.

To model a glaucomatous environment, we also evaluated the effects of 1000 µM bimatoprost and 10 µM BFA on ECM-related gene expression in TM cells treated with TGF-β2 (Fig. 5). Treatment of TM cells with 2.5 ng/mL TGF-β2 for 72 hours led to increased expression of the majority of the genes on the array (see Fig. 5) consistent with results of previous studies (Supplementary Table S8). Co-treatment with 10 µM BFA during the last 24 hours of the treatment period had only minor effects on the levels of gene expression in TGF-β2-treated cells, but co-treatment with 1000 µM bimatoprost substantially modulated TGF-β2-stimulated expression of several genes (see Fig. 5). In TM cells treated with TGF-β2, COL1A1, connective tissue growth factor (CTGF), integrin-alpha 5 (ITGAV), integrin-beta 1 (ITGB1), anosmin-1 (ANOS1), laminin-beta 1 (LAMB1), sarcoglycan-epsilon (SGCE), secreted protein acidic and cysteine rich (SPARC), thrombospondin-1 (THBS1), and thrombospondin 2 (THBS2) gene expression was statistically significantly reduced by co-treatment with 1000 µM bimatoprost, whereas co-treatment with 1000 µM bimatoprost significantly stimulated MMP11 and LAMA3 gene expression levels to 3.57-fold and 7.58-fold, respectively, the levels produced by TFG-β2 alone (Table 5). Although not statistically significant, co-treatment with 1000 µM bimatoprost also resulted in extensive increases in the expression of other MMP genes. The addition of 1000 µM bimatoprost increased MMP1, MMP10, and MMP22 gene expression levels to 11.80-fold, 7.42-fold, and 3.57-fold, respectively, the levels in cells treated with TFG-β2 and vehicle control.

**Table 5.** Statistically Significant (*P* < 0.05) Changes in Gene Expression in TGF-β2–Treated TM Cell Strains Exposed to Bimatoprost and BFA (*n* = 6)

	Mean Fold Gene Expression Relative To TGF-β2 Alone
Gene	1000 µM Bimatoprost Added to TGF-β2
COL1A1	0.30
CTGF	0.43
ITGAV	0.33
ITGB1	0.50
ANOS1	0.45
LAMA3	7.58
LAMB1	0.62
MMP11	3.57
SGCE	0.69
SPARC	0.53
THBS1	0.25
THBS2	0.63

BFA, bimatoprost free acid; TGF-β2, transforming growth factor-beta 2; TM, trabecular meshwork.

**Figure 6.**
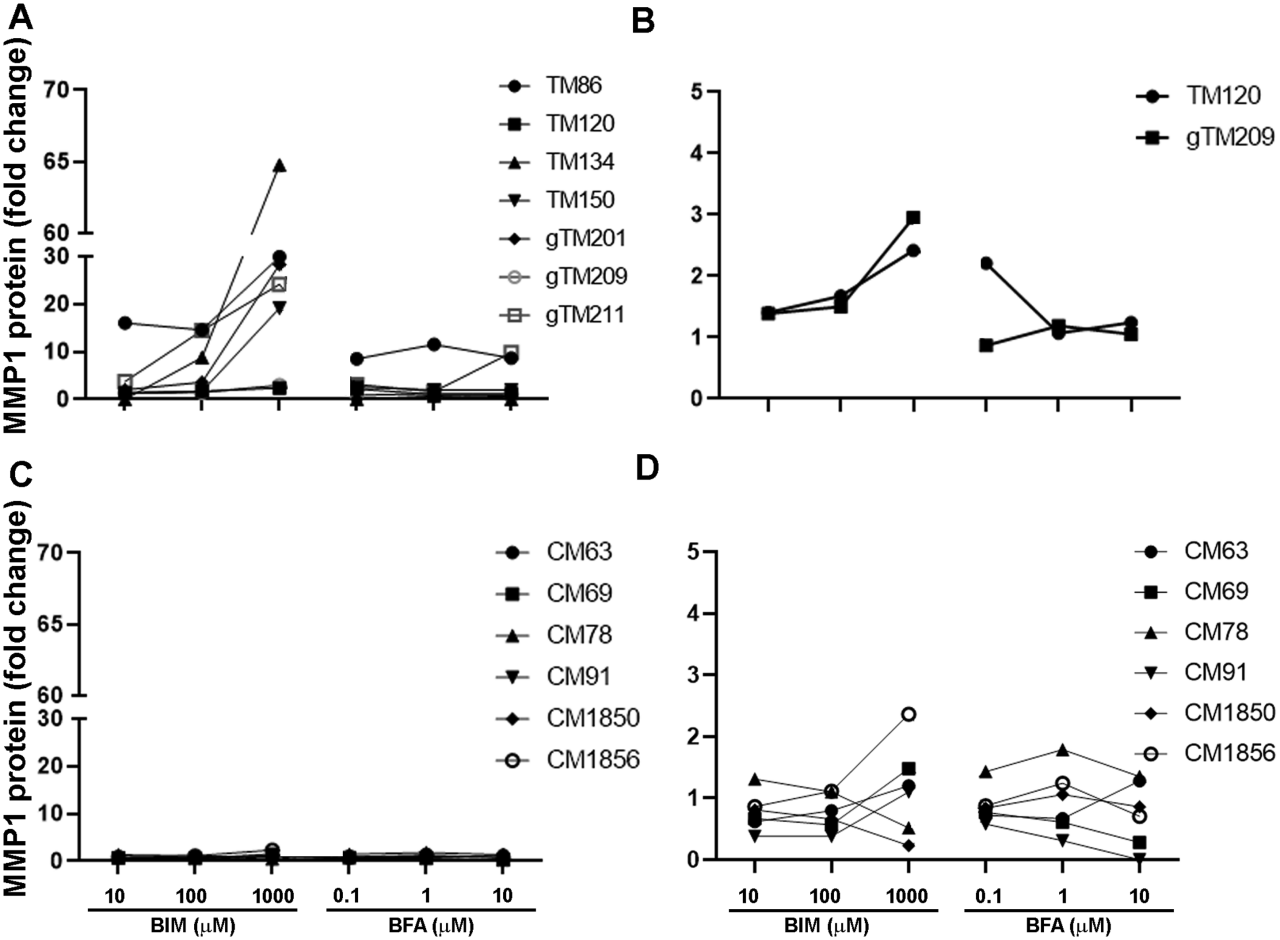
MMP1 protein levels in media conditioned by (**A, B**) trabecular meshwork (TM) cell strains (*n* = 7) and (**C, D**) ciliary muscle (CM) cell strains (*n* = 6) for 24 hours in the presence of bimatoprost (BIM; 10 µM, 100 µM, and 1000 µM) or bimatoprost free acid (BFA; 0.1 µM, 1 µM and 10 µM). Data from 2 TM cell strains **B** and all CM cell strains **D** are shown with an expanded y-axis scale for comparisons. Protein levels are expressed as fold levels relative to vehicle-treated control cells. MMP, matrix metalloproteinase.

Because of the robust effects of bimatoprost on MMP1 gene expression in all TM cell strains tested, MMP1 protein levels were evaluated using media samples conditioned by TM and CM cells treated with pharmacologically relevant concentrations of bimatoprost or BFA, or with vehicle control, for 24 hours. The results were consistent with the results of the gene expression assays. The greatest fold changes in MMP1 protein levels were observed with the 1000 µM concentration of bimatoprost in TM cell cultures (Fig. 6). Bimatoprost stimulated a substantial, concentration-dependent increase in MMP1 secretion from TM cells, with the magnitude of the increase showing variability among the cell strains (see Fig. 6A). The mean increase in MMP1 secretion from TM cells with 1000 µM bimatoprost was 27-fold (*P* = 0.053). In contrast, BFA stimulated MMP1 secretion from TM cells to a much lesser extent, and there was no clear dose-response (see Fig. 6A). Both bimatoprost and BFA also stimulated MMP1 protein secretion from CM cells to a lesser extent (see Fig. 6C).

**Figure 7.**
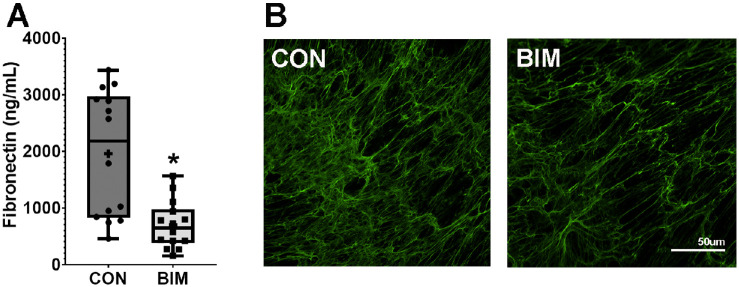
Treatment of human trabecular meshwork (TM) cells with implant levels of bimatoprost reduced fibronectin secretion and deposition. Panel (**A**) shows analysis of fibronectin in conditioned media from 12 different TM cell strains that were treated with bimatoprost (BIM; 1000 µM) or vehicle control (CON) for 24 hours. Panel (**B**) shows immunofluorescence images of fibronectin content in extracellular matrix of confluent TM cells (TM86) treated with or without bimatoprost for 24 hours. Fibronectin content was generally less complex and less abundant in treated samples, but was variable depending upon cell strain examined (Supplementary Fig. S9). The line and cross in the box whisker plots correspond to the median and mean, respectively. The *top* and *bottom* edges of the box indicate the 25th and 75th percentiles, and the whiskers extend to the most extreme data points. *P = 0.00005 versus CON.

To examine the effect of implant levels of bimatoprost on ECM produced by TM cells, secretion and deposition levels of an abundant TM ECM protein, fibronectin, were analyzed. After 24 hours of exposure to bimatoprost, secretion of fibronectin was dramatically reduced by 65% in conditioned media (from 1958.2 +/− 1113.3 ng/mL to 698.5 +/− 422.4 ng/mL, *n* = 14, *P* = 0.00005; Fig. 7). This reduction in secretion corresponded to a reduction in fibronectin abundance in ECM produced by cultured TM cells (see Fig. 7). However, the reduction was highly variable, with some cell strains showing dramatic remodeling and some very little in the 24-hour treatment period (Supplementary Fig. S9).

## Discussion

This study is the first to report the effects of clinically relevant concentrations of bimatoprost and BFA on MMP and ECM gene expression in three different outflow cell types. Although data presented were limited to human cell culture models, this study used the largest number of different cell strains examined to date, including 12 TM cell strains, 6 CM cell strains, and 4 SF cell strains. Previous studies using cultured human cells have reported effects of various PGAs on MMP and ECM gene expression in a limited number of TM and CM cell strains. Here, we included SF cells, as well as TM and CM cells, because both CM and SF cells could contribute to ECM remodeling in the uveoscleral pathway. Exposure of human sclera to various PGAs in organ culture has been shown to cause increased scleral permeability (assessed by movement of rhodamine-dextran across the sclera in a 2-chamber Ussing apparatus) accompanied by increased expression of MMPs.^28^

The concentrations of bimatoprost and BFA tested were based on the dog pharmacokinetic study results for implant and topical treatment extrapolated to humans. As the preclinical study showed very high tissue concentrations of bimatoprost achieved with the implant, and lower concentrations of BFA achieved with both the implant and topical dosing, 3-log-unit ranges of bimatoprost concentrations (10 to 1000 µM) and BFA concentrations (0.1 to 10 µM) were selected for testing. These ranges reflect estimated bimatoprost concentrations that occur after implant treatment of human eyes and estimated BFA concentrations that occur after implant and topical treatment of human eyes.

Previous studies have reported that PGA-induced upregulation of MMPs in TM and CM cells is dependent on both the identity and concentration of the PGA.^18^^–^^21^^,^^29^^,^^30^ Consistent with these findings, we observed that the effects of PGA treatment on the expression of ECM-related genes in cells derived from outflow tissues were dependent on the PGA form (bimatoprost versus BFA), the concentration, and the cell type. Importantly, in all 3 cell types (TM, CM, and SF cells) the 1000 µM concentration of bimatoprost had the most substantial effect on gene expression. For example, MMP14 (MT1-MMP), a type I transmembrane protein whose ECM substrates include fibronectin and laminin, and that also can cleave and activate proMMP2 and proMMP13,^22^ was significantly upregulated by bimatoprost only, and only at the 100 µM concentration, in each cell type. Notably, however, for each cell type, large variability in the response to 1000 µM bimatoprost was observed among cell strains from different donors. The largest observed effect on MMP gene expression was the upregulation of MMP1 by 1000 µM bimatoprost in TM cells, yet the mean fold upregulation did not reach statistical significance because of the variability in response among cell strains. Variability in the bimatoprost-stimulated upregulation in MMPs among cell strains from different donors may help explain differential long-term responses of patients to the bimatoprost implant.

A study by Oh et al.^19^ evaluated the effects of latanoprost free acid treatment on MMP and tissue inhibitor of matrix metalloproteinase (TIMP) gene expression by cultures of TM cells from five human donors. Exposure of the cultures to latanoprost acid at a pharmacologic concentration of 77 nM (i.e., a concentration achieved in the aqueous humor after topical ophthalmic dosing of latanoprost) for 24 hours in serum-free medium resulted in increased expression of mRNA for MMP1 (in 4 of 5 cultures), MMP3 (in 4 of 5 cultures), MMP17 (in 3 of 5 cultures), MMP24 (in all 5 cultures), and TIMPs 2, 3, and 4 (in 3 of 5 cultures), whereas MMP11 (in 4 of 5 cultures) and MMP15 (in 3 of 5 cultures; upregulated in 1 of 5) were downregulated. Consistent with these findings, in our study BFA at a similar concentration of 100 nM (relevant to topical ophthalmic dosing of bimatoprost) increased mean MMP1, MMP3, and TIMP3 gene expression. However, a dramatic 62.9-fold increase in MMP1 gene expression was seen only with the 1000 µM concentration of bimatoprost that can be achieved with bimatoprost implant treatment. In addition, in our study, 100 nM BFA produced no substantial decrease in mean MMP11 and MMP15 gene expression, and rather than causing downregulation of MMP11 gene expression, 1000 µM bimatoprost increased mean MMP11 gene expression, as well as MMP11 gene expression by each individual TM cell strain tested.

Several previous studies have evaluated the effects of PGAs on MMP and TIMP gene expression by CM cells. In a study by Weinreb et al.^29^ using cultures of CM cells isolated from eyes of 8 human donors, latanoprost acid (tested at concentrations of 50 nM to 1 µM) upregulated gene expression of MMP1 (in 5 of 5 cultures), MMP3 (in 3 of 5 cultures), and MMP9 (in 4 of 5 cultures), and decreased MMP2 gene expression (in 3 of 5 cultures), compared with the vehicle control. Another study from the same laboratory showed that TIMP1 gene expression was upregulated in cultures of human CM cells after treatment with 1 µM or 10 µM latanoprost acid for 18 hours.^17^ In an additional study by Oh et al.^26^ using cultures of CM cells isolated from human donor eyes, treatment with 77 nM latanoprost acid for 24 hours stimulated MMP9 gene expression and increased gene expression of MMP3 (in 3 of 5 cultures), MMP17 (in 4 of 5 cultures), and TIMP3 (in all 5 cultures), and decreased gene expression of MMP1 (in all 5 cultures), MMP2 (in 3 of 5 cultures), and TIMP4 (in 2 of 5 cultures).^26^ The reasons for the difference in the effect of latanoprost acid treatment on MMP1 gene expression in the previously reported studies (upregulation in the study by Weinreb et al.^29^ and downregulation in the study by Oh et al.^26^) are unexplained but could involve differences in the cell strains, the cell culture and treatment conditions (including the vehicle and the presence of serum), and the primers and methods used for the quantitation of gene expression (real-time PCR versus quantitative reverse transcription PCR, respectively). By comparison, in our study, 100 nM BFA slightly downregulated and 1000 µM bimatoprost increased mean MMP1 gene expression relative to the control. Consistent with the reported effects of latanoprost acid on MMP2 gene expression by CM cells, our results showed that both 100 nM BFA and 1000 µM bimatoprost decreased mean MMP2 gene expression. However, neither 100 nM BFA nor 1000 µM bimatoprost increased MMP3 expression by CM cells, and only 1000 µM bimatoprost, not 100 nM BFA, upregulated mean MMP9 and TIMP1 gene expression by CM cells. Similar to latanoprost acid, 100 nM BFA increased TIMP3 gene expression by CM cells, whereas 1000 µM bimatoprost did not.

PGA effects on gene expression in cultures of human SF cells have not been studied extensively. However, a study using human scleral organ/explant cultures showed that 100 nM PGF_2α_ or latanoprost acid upregulated gene expression for MMPs 1, 3, 9, and 10 as well as TIMPs 1, 2, and 3.^18^ Similar to these findings, in our study, both 100 nM BFA and 1000 µM bimatoprost increased mean MMP1 and MMP3 gene expression in SF cells. However, 100 nM BFA and 1000 µM bimatoprost either had no effect on, or decreased, mean MMP9, MMP10, TIMP1, TIMP2, and TIMP3 gene expression in SF cells.

Previous studies have suggested that the balance of expression of MMPs and TIMPs may be altered in patients in POAG, leading to decreased outflow facility and increased IOP.^31^^,^^32^ Because MMP gene expression could potentially be differentially regulated in eyes with glaucoma, we tested the effects of bimatoprost and BFA on TM cells from eyes with glaucoma, as well as on cells from normal eyes. The pattern of changes in gene expression produced by 1000 µM bimatoprost appeared to be similar in TM cells from glaucomatous eyes and TM cells from normal eyes. However, the mean fold upregulation of MMP1 mRNA was even greater in the cells from glaucomatous eyes, reaching statistical significance. Heat maps of mean gene expression also showed an apparent increase in the number of genes upregulated by 10 µM BFA in cells from glaucomatous eyes compared with cells from normal eyes, but the changes in expression levels of these genes were generally not statistically significant.

The reasons for the accumulation of ECM proteins in the TM and the subsequent decrease in outflow facility in eyes with POAG are not fully understood. However, TGF-β2 is thought to have a major role, because aqueous humor levels of TGF-β2 are elevated in POAG,^33^ and perfusion of TGF-β2 in cultured human anterior segments has been shown to cause ECM deposits and increase outflow resistance.^34^ Therefore, in some experiments, TM cells were treated with 2.5 ng/mL human recombinant TGF-β2 to mimic glaucomatous conditions. At the concentrations tested, bimatoprost had substantial effects, and BFA had minor effects, on the expression of ECM-related genes in the TGF-β2–treated TM cells. Of note, TGF-β2 upregulated MMP11 in the TM cells, and co-treatment with 1000 µM bimatoprost statistically significantly upregulated MMP11 gene expression over the level produced by TGF-β2 alone. Bimatoprost at a 1000 µM concentration also markedly increased MMP1, MMP10, and MMP22 gene expression levels over those observed in cells treated with TGF-β2 and vehicle control. These results suggest that a concentration of bimatoprost relevant to bimatoprost implant administration could cause extensive TM tissue remodeling in glaucomatous eyes.

There is ample evidence that MMP1 is involved in the effects of PGAs on aqueous humor outflow. MMP1 is present in tissues associated with the uveoscleral outflow pathway in normal human eyes (ciliary muscle, iris root, and sclera), suggesting that MMP1 activity may regulate uveoscleral outflow.^35^ In addition, studies in cynomolgus monkeys have shown that topical ophthalmic PGA treatment leads to tissue remodeling and loss of ECM in the ciliary body and TM,^14^^,^^16^^,^^36^ and is associated with both decreased IOP and significantly increased MMP1 immunoreactivity in tissues associated with uveoscleral outflow.^15^ Importantly, in a pharmacogenetic study involving 117 Spanish patients with open-angle glaucoma, 6 subhaplotypes of the MMP1 gene were associated with lack of response to latanoprost,^37^ strongly implicating MMP1 in the mechanism of IOP lowering with PGAs.

Expression of MMPs is known to be transcriptionally regulated,^38^ and assays of MMP1 protein levels in the conditioned medium of bimatoprost- and BFA-treated TM and CM cells confirmed the upregulation of MMP1 protein secretion as well as MMP1 gene expression. Consistent with the gene expression assays, effects on protein levels were variable among the cell strains from different donors, and the 1000 µM concentration of bimatoprost had the greatest effect, stimulating a mean 27-fold increase in MMP1 protein levels in the conditioned medium of TM cells. The increase in MMP1 protein expression was dose-dependent and much larger in the TM cell cultures than in the CM cell cultures, suggesting the possibility that remodeling of the conventional outflow pathway may be most important in the long-term IOP lowering with the bimatoprost implant. Consistent with this possibility, we observed a decrease in fibronectin secretion and deposition in the ECM of bimatoprost-treated TM cells. Another possibility is that lymphangiogenesis may be involved in the long-term IOP lowering with the bimatoprost implant. A study in cynomolgus monkeys showed that PGA treatment for 1 year produced empty spaces lined with endothelial-like cells between muscle bundles in the ciliary muscle.^16^ We have hypothesized that these endothelial cell–lined spaces induced by PGAs function as new lymphatic vessels, and persistence of these endothelial-lined channels may explain the extended IOP lowering observed with the bimatoprost implant (Rhee D, presented at the Cleveland Eye Bank Foundation Second Annual Virtual Vision Research Symposium, February 15, 2022).

In summary, in this study, bimatoprost and BFA had differential effects on MMP gene expression by cells cultured from human outflow tissues. A dramatic upregulation of MMP1 gene expression by TM and CM cells was seen only with intact bimatoprost, and only at the high drug levels observed in bimatoprost implant–treated eyes. Variability in the bimatoprost-stimulated upregulation in MMPs among cell strains from different donors may help explain differential long-term responses of patients to the bimatoprost implant. Upregulation of MMP1 and decreased fibronectin produced by high bimatoprost concentrations in target tissues after bimatoprost implant administration may lead to sustained tissue remodeling and long-term IOP reduction in the absence of drug.

## Supplementary Material

Supplement 1
