# Supplementary material for: Proposed Mechanism of Long-Term Intraocular Pressure Lowering With the Bimatoprost Implant
Source: Invest Ophthalmol Vis Sci. 2023 Mar 6;64(3):15. doi: 10.1167/iovs.64.3.15 (PMC10007903; doi:10.1167/iovs.64.3.15)
Supplement: Supplement 1 [file iovs-64-3-15_s001.pdf]

Supplementary Table S1. Characteristics of Cell Strains and Donor Eyes

Cell Strain	Cell Type	Donor				
		Age	Sex	Race/Ethnicity	Ocular Diagnosis	Outflow Facility (µL/min/mmHg)
gTM201	Trabecular meshwork	81 y	Female	White	POAG	0.13
gTM209	Trabecular meshwork	71 y	Male	White	POAG	0.06
gTM211	Trabecular meshwork	75 y	Female	White	POAG	0.17
TM86	Trabecular meshwork	3 m	N/A	N/A	Normal	nd
TM96	Trabecular meshwork	28 y	Male	White	Normal	nd
TM120	Trabecular meshwork	11 m	Male	N/A	Normal	nd
TM129	Trabecular meshwork	75 y	Female	White	Normal	nd
TM134	Trabecular meshwork	51 y	Male	White	Normal	nd
TM150	Trabecular meshwork	4 m	Female	Black	Normal	nd
TM151	Trabecular meshwork	40 y	Female	Asian	Normal	nd
TM155	Trabecular meshwork	58 y	Female	White	Normal	nd
TM213	Trabecular meshwork	81 y	Male	White	Normal	nd
CB63	Ciliary muscle	63 y	Female	White	Normal	nd
CB69	Ciliary muscle	69 y	Male	White	Normal	nd
CB78	Ciliary muscle	78 y	Female	White	Normal	nd
CB91	Ciliary muscle	91 y	Male	White	Normal	nd
CB1850	Ciliary muscle	76 y	Male	White	Normal	nd
CB1856	Ciliary muscle	79 y	Female	White	Normal	nd
F19001379	Scleral fibroblast	65 y	Female	Black	Normal	nd
F19002086	Scleral fibroblast	52 y	Female	Black	Normal	nd
F19002087	Scleral fibroblast	60 y	Male	White	Normal	nd
F20000019	Scleral fibroblast	66 y	Female	Black	Normal	nd

N/A = not available; nd = not determined; POAG = primary open-angle glaucoma.

Supplementary Table S2. Eighty-four Extracellular Matrix–Related Genes Represented on the Qiagen Array, Identified by Number/Position on the Array

Position	Symbol	Description	Position	Symbol	Description
1	ADAMTS1	ADAM metallopeptidase with thrombospondin type 1 motif, 1	43	ITGB4	Integrin, beta 4
2	ADAMTS13	ADAM metallopeptidase with thrombospondin type 1 motif, 13	44	ITGB5	Integrin, beta 5
3	ADAMTS8	ADAM metallopeptidase with thrombospondin type 1 motif, 8	45	ANOS1	Kallmann syndrome 1 sequence
4	CD44	CD44 molecule (Indian blood group)	46	LAMA1	Laminin, alpha 1
5	CDH1	Cadherin 1, type 1, E-cadherin (epithelial)	47	LAMA2	Laminin, alpha 2
6	CLEC3B	C-type lectin domain family 3, member B	48	LAMA3	Laminin, alpha 3
7	CNTN1	Contactin 1	49	LAMB1	Laminin, beta 1
8	COL11A1	Collagen, type XI, alpha 1	50	LAMB3	Laminin, beta 3
9	COL12A1	Collagen, type XII, alpha 1	51	LAMC1	Laminin, gamma 1 (formerly LAMB2)
10	COL14A1	Collagen, type XIV, alpha 1	52	MMP1	Matrix metallopeptidase 1 (interstitial collagenase)
11	COL15A1	Collagen, type XV, alpha 1	53	MMP10	Matrix metallopeptidase 10 (stromelysin 2)
12	COL16A1	Collagen, type XVI, alpha 1	54	MMP11	Matrix metallopeptidase 11 (stromelysin 3)
13	COL1A1	Collagen, type I, alpha 1	55	MMP12	Matrix metallopeptidase 12 (macrophage elastase)
14	COL4A2	Collagen, type IV, alpha 2	56	MMP13	Matrix metallopeptidase 13 (collagenase 3)
15	COL5A1	Collagen, type V, alpha 1	57	MMP14	Matrix metallopeptidase 14 (membrane-inserted)
16	COL6A1	Collagen, type VI, alpha 1	58	MMP15	Matrix metallopeptidase 15 (membrane-inserted)
17	COL6A2	Collagen, type VI, alpha 2	59	MMP16	Matrix metallopeptidase 16 (membrane-inserted)
18	COL7A1	Collagen, type VII, alpha 1	60	MMP2	Matrix metallopeptidase 2 (gelatinase A)
19	COL8A1	Collagen, type VIII, alpha 1	61	MMP3	Matrix metallopeptidase 3 (stromelysin 1, progelatinase)
20	CTGF	Connective tissue growth factor	62	MMP7	Matrix metallopeptidase 7 (matrilysin, uterine)
21	CTNNA1	Catenin (cadherin-associated protein), alpha 1, 102kDa	63	MMP8	Matrix metallopeptidase 8 (neutrophil collagenase)
22	CTNNB1	Catenin (cadherin-associated protein), beta 1, 88kDa	64	MMP9	Matrix metallopeptidase 9 (gelatinase B)
23	CTNND1	Catenin (cadherin-associated protein), delta 1	65	NCAM1	Neural cell adhesion molecule 1
24	CTNND2	Catenin (cadherin-associated protein), delta 2	66	PECAM1	Platelet/endothelial cell adhesion molecule
25	ECM1	Extracellular matrix protein 1	67	SELE	Selectin E
26	FN1	Fibronectin 1	68	SELL	Selectin L
27	HAS1	Hyaluronan synthase 1	69	SELP	Selectin P (granule membrane protein 140kDa, antigen CD62)
28	ICAM1	Intercellular adhesion molecule 1	70	SGCE	Sarcoglycan, epsilon
29	ITGA1	Integrin, alpha 1	71	SPARC	Secreted protein, acidic, cysteine-rich (osteonectin)
30	ITGA2	Integrin, alpha 2 (CD49B, alpha 2 subunit of VLA-2 receptor)	72	SPG7	Spastic paraplegia 7 (pure and complicated autosomal recessive)
31	ITGA3	Integrin, alpha 3	73	SPP1	Secreted phosphoprotein 1
32	ITGA4	Integrin, alpha 4	74	TGFBI	Transforming growth factor, beta-induced, 68kDa
33	ITGA5	Integrin, alpha 5 (fibronectin receptor, alpha polypeptide)	75	THBS1	Thrombospondin 1
34	ITGA6	Integrin, alpha 6	76	THBS2	Thrombospondin 2
35	ITGA7	Integrin, alpha 7	77	THBS3	Thrombospondin 3
36	ITGA8	Integrin, alpha 8	78	TIMP1	TIMP metallopeptidase inhibitor 1
37	ITGAL	Integrin, alpha L	79	TIMP2	TIMP metallopeptidase inhibitor 2
38	ITGAM	Integrin, alpha M (complement component 3 receptor 3 subunit)	80	TIMP3	TIMP metallopeptidase inhibitor 3
39	ITGAV	Integrin, alpha V (vitronectin receptor)	81	TNC	Tenascin C
40	ITGB1	Integrin, beta 1 (fibronectin receptor)	82	VCAM1	Vascular cell adhesion molecule 1
41	ITGB2	Integrin, beta 2	83	VCAN	Versican
42	ITGB3	Integrin, beta 3 (platelet glycoprotein IIIa, antigen CD61)	84	VTN	Vitronectin

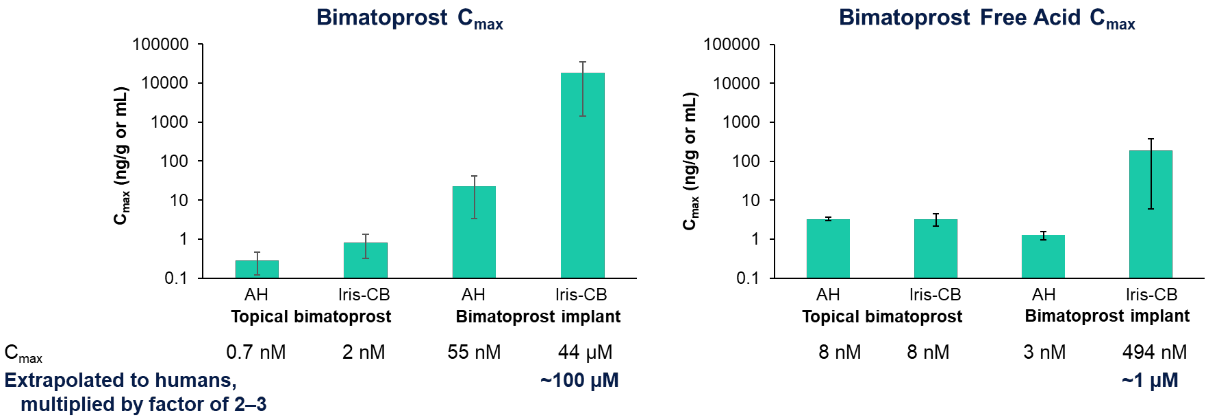

Supplementary Figure S3. Maximal drug concentration (C_{max}) for (left) bimatoprost and (right) bimatoprost free acid in target tissues for intraocular pressure lowering after intracameral administration of a 15-µg bimatoprost implant or topical administration of bimatoprost 0.03% once daily for 7 days in beagle dogs (n=4 eyes [2 dogs] per timepoint). Data shown are mean ± standard error of the mean. Estimations of mean C_{max} values for bimatoprost and bimatoprost free acid that could be achieved in the iris-CB after administration of the bimatoprost implant in humans, extrapolated from the data in dogs, are shown in boldface type below the graphs. AH = aqueous humor; CB = ciliary body.

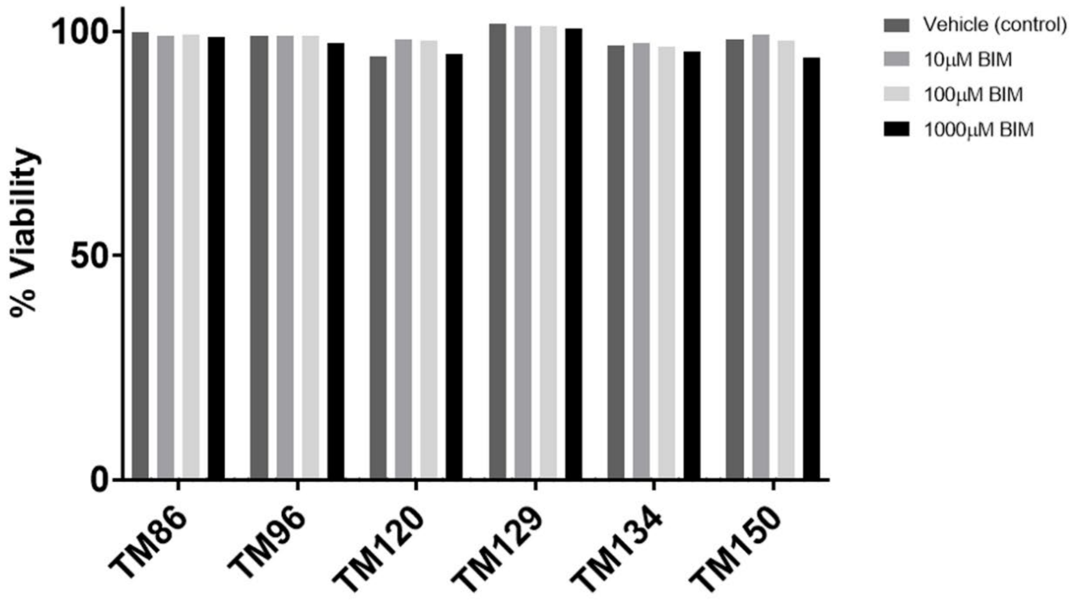

Supplementary Figure S4. Viability of trabecular meshwork (TM) cell strains (n=6) after treatment for 24 hours with a bimatoprost implant–relevant concentration of bimatoprost (BIM; 10 μM, 100 μM, or 1000 μM) or the vehicle control (1% EtOH).

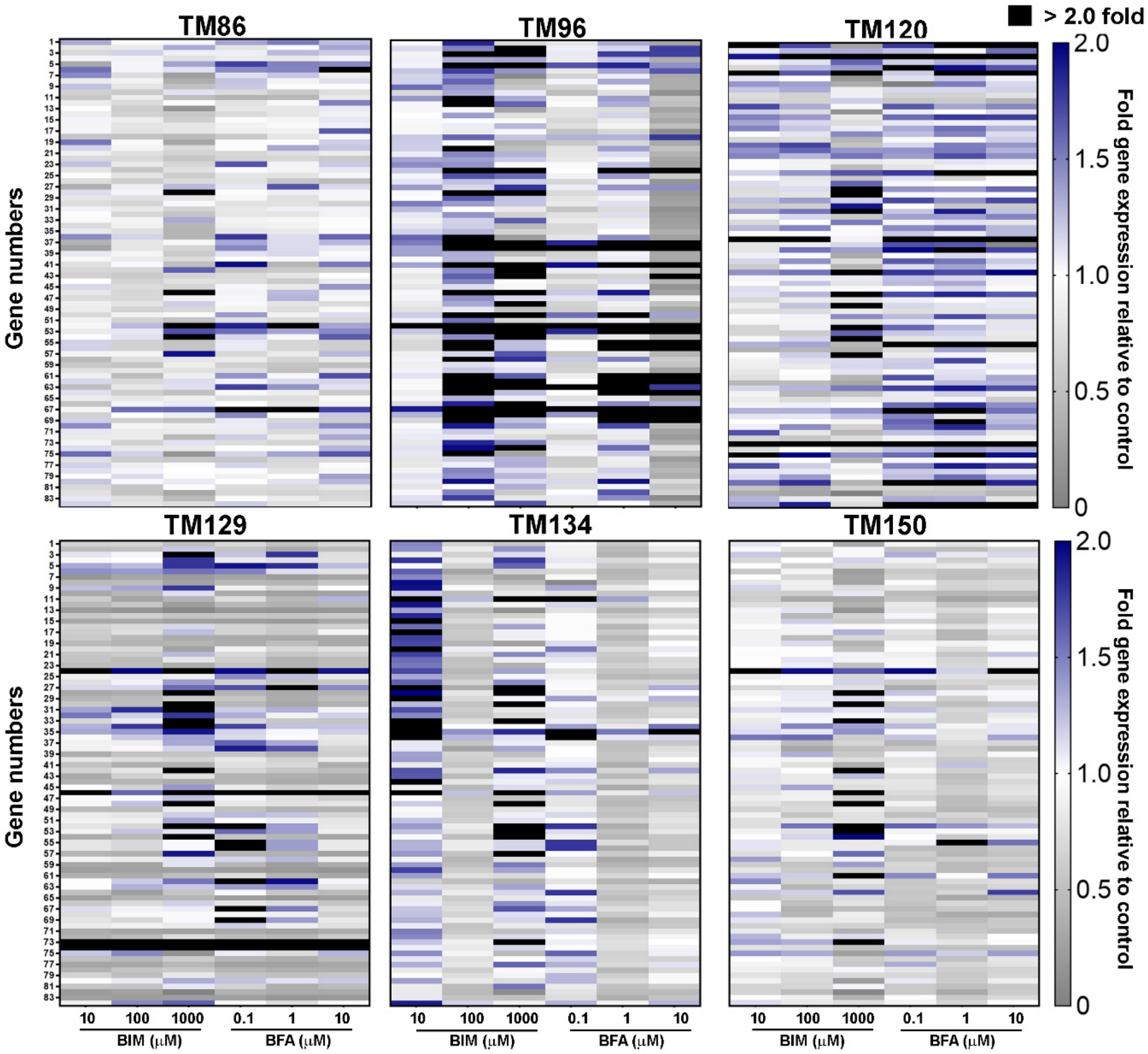

Supplementary Figure S5. Heat map of the expression levels of 84 extracellular matrix–related genes in trabecular meshwork (TM) cell strains (n=6) in response to treatment for 24 hours with bimatoprost implant– or topical bimatoprost–relevant concentrations of bimatoprost (BIM; 10 μM, 100 μM, or 1000 μM) or bimatoprost free acid (BFA; 0.1 μM, 1 μM, or 10 μM), respectively, relative to control (vehicle) treatment. Expression levels are indicated by colors, where grays indicate a decrease, white indicates no change, and blue and black indicate an increase in gene expression relative to control.

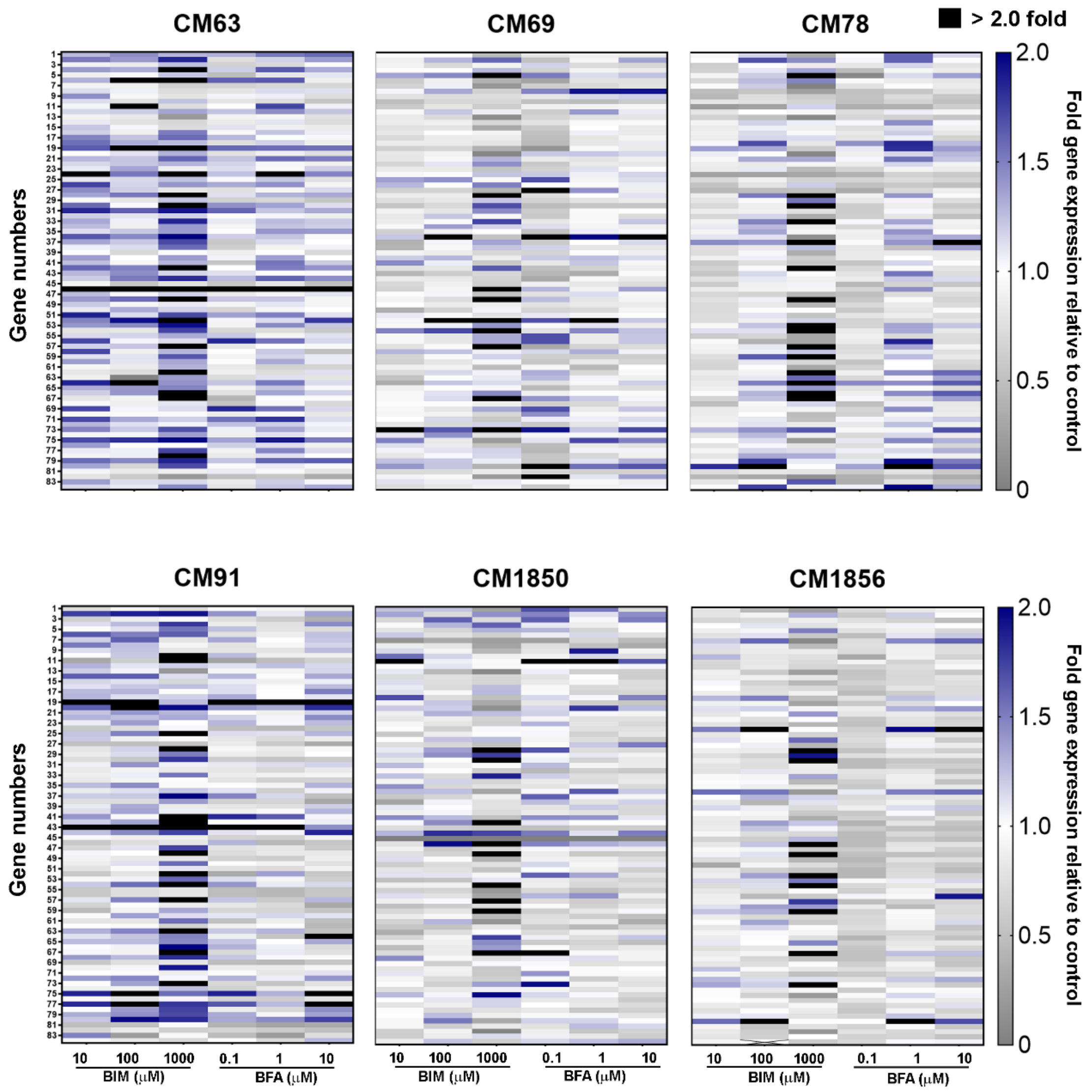

Supplementary Figure S6. Heat map of the expression levels of 84 extracellular matrix–related genes in ciliary muscle (CM) cell strains (n=6) in response to treatment for 24 hours with bimatoprost implant– or topical bimatoprost–relevant concentrations of bimatoprost (BIM; 10 μM, 100 μM, or 1000 μM) or bimatoprost free acid (BFA; 0.1 μM, 1 μM, or 10 μM), respectively, relative to control (vehicle) treatment. Expression levels are indicated by colors, where grays indicate a decrease, white indicates no change, and blue and black indicate an increase in gene expression relative to control.

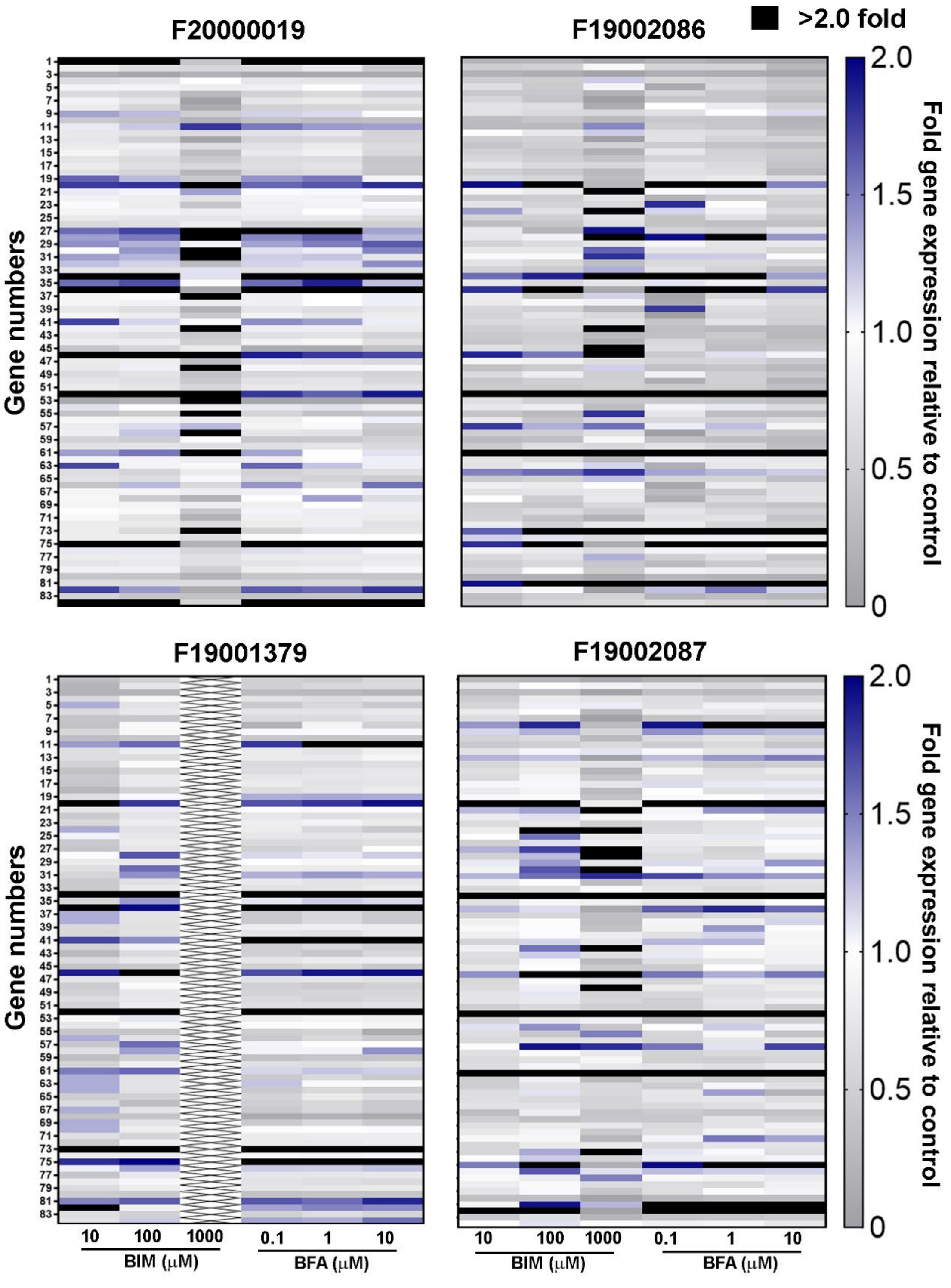

Supplementary Figure S7. Heat map of the expression levels of 84 extracellular matrix–related genes in scleral fibroblast cell strains (n=4) in response to treatment for 24 hours with bimatoprost implant– or topical bimatoprost–relevant concentrations of bimatoprost (BIM; 10 μM, 100 μM, or 1000 μM) or bimatoprost free acid (BFA; 0.1 μM, 1 μM, or 10 μM), respectively, relative to control (vehicle) treatment. Expression levels are indicated by colors, where grays indicate a decrease, white indicates no change, and blue and black indicate an increase in gene expression relative to control.

Supplementary Table S8. Genes Previously Shown to be Upregulated in Response to TGF-β2 Treatment of TM Cells[1-5] and Results in 4 TM Cell Strains Treated With TGF-β2 in the Present Study

Affected Gene	Fold Gene Expression With 2.5 ng/mL TGF-β2 Treatment Relative to Vehicle Control Treatment			
	TM120	TM134	TM96	TM129
Collagen type I alpha 1 (COL1A1)	8.65	24.72	2.31	8.08
Collagen type V alpha 1 (COL5A1)	8.77	10.91	11.28	8.13
Fibronectin 1 (FN1)	2.09	1.92	1.68	5.37
Thrombospondin 1 (THBS1)	5.36	5.65	6.26	3.08
Versican (CSPG2)	27.7	321.24	15.52	19.32
Hyaluronan synthase 1 (HAS1)	30.53	36.95	4.65	3.06
Secreted protein acidic and cysteine rich (SPARC)	6.46	8.27	6.8	4.36
Matrix metalloproteinase 2 (MMP2)	2.64	3.8	3.15	8.84

TGF-β2, transforming growth factor-beta 2; TM, trabecular meshwork.

1. Fleenor DL, Shepard AR, Hellberg PE, Jacobson N, Pang IH, Clark AF. TGFbeta2-induced changes in human trabecular meshwork: implications for intraocular pressure. *Invest Ophthalmol Vis Sci*. 2006;47(1):226–234.

2. Zhao X, Ramsey KE, Stephan DA, Russell P. Gene and protein expression changes in human trabecular meshwork cells treated with transforming growth factor-beta. *Invest Ophthalmol Vis Sci*. 2004;45(11):4023–4034.

3. Fuchshofer R, Welge-Lussen U, Lütjen-Drecoll E. The effect of TGF-beta2 on human trabecular meshwork extracellular proteolytic system. *Exp Eye Res*. 2003;77(6):757–765.

4. Han H, Kampik D, Grehn F, Schlunck G. TGF-β2-induced invadosomes in human trabecular meshwork cells. *PLoS One*. 2013;8(8):e70595.

5. Kang MH, Oh DJ, Kang JH, Rhee DJ. Regulation of SPARC by transforming growth factor β2 in human trabecular meshwork. *Invest Ophthalmol Vis Sci*. 2013;54(4):2523–2532.

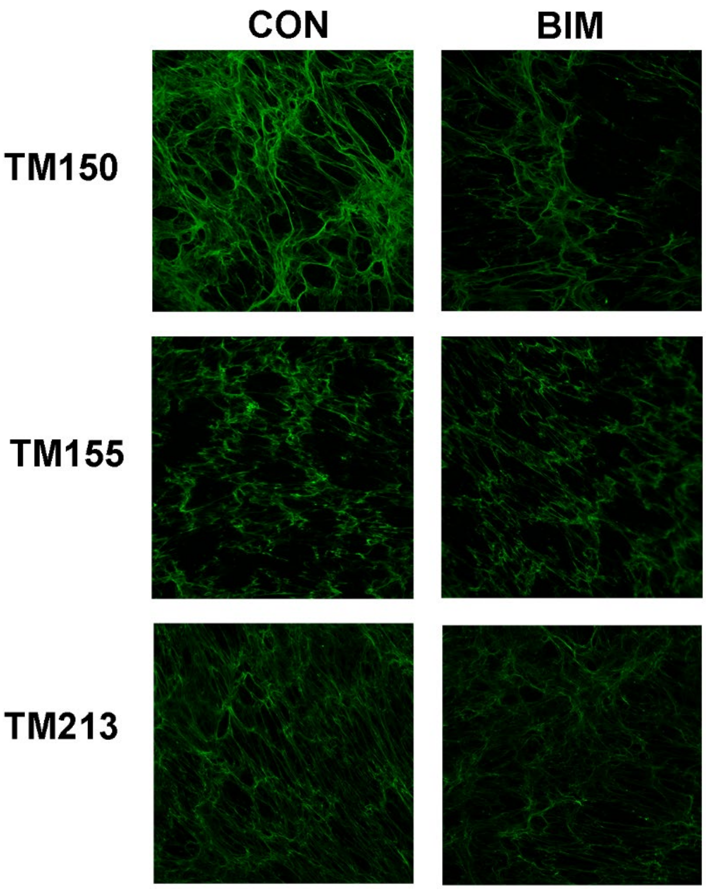

Supplementary Figure S9. Treatment of human trabecular meshwork cells with implant levels of bimatoprost (BIM, 1000 μM) reduce fibronectin deposition to different degrees, depending upon the cell strain examined. Shown are representative immunofluorescence microscopic images of three different trabecular meshwork strains tested. The fourth cell strain tested is shown in **Fig 7**. CON, vehicle control.
